# The dimension and morphology of alveolar bone at maxillary anterior teeth in periodontitis: a retrospective analysis—using CBCT

**DOI:** 10.1038/s41368-019-0071-0

**Published:** 2020-01-14

**Authors:** Xue Zhang, Yuchao Li, Ziming Ge, Haijiao Zhao, Lei Miao, Yaping Pan

**Affiliations:** Department of Periodontics, School of Stomatology, China Medical University, Shenyang, China

**Keywords:** Periodontitis, Oral manifestations

## Abstract

The morphology of the alveolar bone at the maxillary anterior teeth in periodontitis patients was evaluated by cone-beam computed tomography (CBCT) to investigate the distribution of alveolar defects and provide guidance for clinical practice. Ninety periodontitis patients and 30 periodontally healthy individuals were selected to determine the morphology of the alveolar bone at the maxillary anterior teeth according to the degree of bone loss, tooth type, sex and age. The differences in the dimensions between periodontitis patients and healthy individuals were compared, and the distribution of alveolar bone defects was analyzed. A classification system was established regarding the sagittal positions and angulations of the teeth. The buccal residual bone was thicker and the lingual bone was thinner in the periodontitis patients than in the periodontally healthy individuals, and there were differences between the different tooth types, sexes and age subgroups. The buccal undercut was close to the alveolar ridge, while fenestration was reduced and the apical bone height was higher in periodontitis patients than in periodontally healthy individuals. The apical bone height increased with the aggravation of bone loss and age. The proportions of different sagittal positions changed with the aggravation of bone loss. Moreover, the teeth moved more buccally regarding the positions of the maxillary anterior teeth. The morphology of the alveolar bone at the maxillary anterior teeth differed between periodontitis patients and healthy individuals, and the differences were related to the degree of bone loss, tooth type, sex and age.

## Introduction

Periodontitis is a chronic host-mediated inflammatory disease characterised by plaque biofilm contamination that leads to alveolar bone loss.^1^ The consensus report of the 2017 Classification World Workshop emphasised that the degree of alveolar bone loss has been used as direct evidence of the severity and progression of periodontitis.^2^ Clinical bone loss differs based on the patient’s age, tooth type and level of oral biofilm contamination, which may lead to transformations in the morphology of residual bone. With the increasing level of acceptance of periodontal aesthetic surgery, implantation, orthodontics and restorative therapy after the initial therapy, the morphology of alveolar bone defects in periodontitis has attracted more attention.

The maxillary anterior region is becoming a major concern due to its aesthetic relevance. Regardless of whether implantation, orthodontics or restorative therapy is used, the morphology of the alveolar bone is of great importance. Alveolar morphology is associated with regional and ethnic differences, influenced by occlusions and related to facial skeletal types and periodontal biotypes. In the existing literatures, cone-beam computed tomography (CBCT) had been used to study the alveolar bone morphology of the upper anterior area of periodontally healthy people. The common indicators included buccal or palatal bone thickness, the location and depth of undercut and apical bone height.^3–5^ Several studies noted that the buccal bone thickness should be at least ≥2 mm to maintain the alveolar bone level.^6–8^ A thinner buccal bone and the occurrence of undercut may increase the risk of fenestration, soft-tissue recession and cortical bone perforation occurring during or after implantation.^9,10^ Adequate apical bones may influence primary stability by placing the implant deeper apically. The sagittal root position in the alveolar process is classified by the bone thickness and the direction of the root, providing a reference to help avoid bone perforation during implant placement. Besides, the intersection angle between the long axis of the teeth and the alveolar could influence the morphology of alveolar bone. There may be some changes in the morphology of the alveolar bone in periodontitis, but few studies have mentioned this issue.

In the two-dimensional imaging era, intraoral radiography, bitewing radiography and panoramic radiography play important roles in periodontal diagnoses; however, these methods can only measure bone loss in mesial and distal sites, so the understanding of bone loss has considerable limitations. Besides, due to the projection, measurement errors, and anatomical overlap, three-dimensional anatomy of alveolar bone could not be entirely exhibited. Recently, CBCT has been considered a practical device, as it provides three-dimensional images as well as arbitrary levels of data with higher resolution and lower radiation exposure than the other methods. Zhao et al.^11^ summarized the patterns of alveolar bone defects in periodontitis using CBCT, finding that it was tooth type and site specific. A large number of studies confirmed that CBCT can accurately evaluate the loss of alveolar bone.^12–15^ Vandemberghe et al.^14^ found that intraoral radiography provided more bone details, including laminar dura and contrast ratio. However, CBCT exhibited more morphological details in bone defects, including furcation involvement, undercut and fenestration.

The objectives of this study were to measure the morphology of the maxillary alveolar bone in periodontitis patients and evaluate the differences in the dimensions between periodontitis patients and healthy individuals in order to investigate the distribution of alveolar bone defects and provide guidance for clinical practice.

## Results

### Differences in morphology between healthy individuals and periodontitis patients

For the periodontitis patients and healthy individuals, the overall buccal residual bone thicknesses were (1.27 ± 0.42) mm (95%CI: 1.27–1.31) and (1.05 ± 0.35) mm (95%CI: 1.00–1.10), respectively, while the palatal thicknesses were (4.05 ± 1.12) mm (95%CI: 3.96–4.15) and (4.46 ± 1.54) mm (95%CI: 4.24–4.69), respectively. Compared with healthy individuals, periodontitis patients had a buccal undercut that was closer to the alveolar crest, but fenestration was relatively rare. A significantly smaller angulation (15.96° ± 6.41°) (95%CI: 15.41–16.50) and larger apical bone height ((11.95 ± 3.37) mm) (95%CI: 11.66–12.24) were measured in periodontitis patients compared with healthy individuals (Appendix Table 1).

### Differences in morphology according to the severity of alveolar bone loss

The buccal residual bone thickness increased significantly when the alveolar bone loss was å 1/2 of the root length optionally, while the palatal residual bone decreased when the bone loss was å 1/3 of the root length optionally. In the severe group, 56.6% of the teeth had a buccal undercut, which was a lower percentage than those of the no bone loss group (67.8%), mild bone loss group (67.3%) and moderate bone loss group (71.4%). Moreover, the undercut was closer to the alveolar ridge in the severe group than in the other groups. The proportion of fenestration gradually decreased in the moderate and severe groups. The angulation in the severe bone loss group was 14.43° ± 6.28° (95%CI: 13.40–15.47), which was significantly smaller than those in the no bone loss group (17.21°  ± 6.90°) (95%CI: 16.20–18.23), mild bone loss group (16.18°  ± 5.89°) (95%CI: 15.35–17.01) and moderate bone loss group (16.85°  ± 6.83°) (95%CI: 15.89–17.81). The apical bone heights in the three bone loss groups were higher than that in the no bone loss group, and it was the highest in the severe group ((12.58 ± 3.74) mm) (95%CI: 11.96–13.19) (Table 1).

**Table 1:** Alveolar bone morphology measurements among different degrees of bone loss.

Alveolar bone morphology	Severity of alveolar bone loss				*P*-value
	No bone loss (*n* = 180)	Mild bone loss (*n* = 196)	Moderate bone loss (*n* = 196)	Severe bone loss (*n* = 143)	
Buccal bone thickness/mm	1.05 ± 0.35	1.20 ± 0.37	1.22 ± 0.40	1.42 ± 0.47	<0.001^a^*
Palatal bone thickness/mm	4.46 ± 1.54	4.25 ± 1.14	3.95 ± 1.12	3.92 ± 1.04	0.001^a†^
Buccal undercut/%	112/180 (67.8%)	132/196 (67.3%)	140/196 (71.4%)	81/143 (56.6%)	0.029^b^*
Buccal undercut depth/mm	1.56 ± 0.65	1.76 ± 0.81	1.72 ± 0.70	1.72 ± 0.82	0.264
Buccal undercut location/mm	5.85 ± 1.46	5.40 ± 1.75	5.30 ± 1.50	4.93 ± 1.43	<0.001^a‡^
Buccal fenestration/%	87/180 (48.3%)	99/196 (50.5%)	62/196 (31.6%)	16/143 (11.1%)	<0.001^b†^
Angulation between long axis of teeth and alveolar process/°	17.21 ± 6.90	16.18 ± 5.89	16.85 ± 6.83	14.43 ± 6.28	0.004^a§^
Apical bone height/mm	10.17 ± 3.17	11.41 ± 3.35	12.03 ± 3.02	12.58 ± 3.74	<0.001^a‖^

^a^Kruskal–Wallis test among different degrees of bone loss

^a*^Buccal residual bone thickness, no bone loss group versus mild, moderate, and severe bone loss groups, *P* < 0.05; mild versus severe loss group, *P* < 0.05; moderate versus severe loss group, *P* < 0.05

^a†^Palatal residual bone thickness, no bone loss group versus moderate and severe bone loss groups, *P* < 0.05; mild bone loss group versus moderate and severe bone loss groups, *P* < 0.05

^a‡^Buccal undercut location, no bone loss group versus severe bone loss group, *P* < 0.05

^a§^Angle, severe bone loss group versus no bone loss, mild bone loss, and moderate bone loss groups, *P* < 0.05

^a‖^Apical bone height, no bone loss group versus mild, moderate and severe bone loss groups, *P* < 0.05; mild versus severe loss group, *P* < 0.05

^b^Chi-square test among different degrees of bone loss

^b*^Percentage of buccal undercut, severe bone loss group versus no bone loss, mild bone loss, and moderate bone loss groups, *P* < 0.05

^b†^Percentage of buccal fenestration, moderate and severe bone loss groups versus no bone loss and mild bone loss groups, *P* < 0.05; moderate versus severe loss group, *P* < 0.05

### Differences in alveolar bone morphology by tooth type, sex and age in patients with periodontitis

#### Tooth type and site differences

**Fig. 1:**
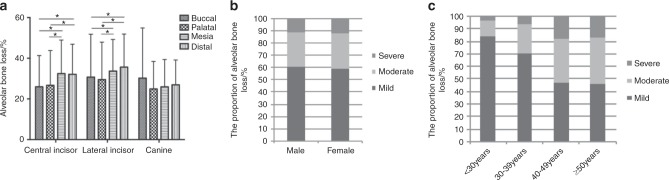
Differences in alveolar bone morphology by tooth type, sex and age in patients with periodontitis. **a** Distribution of alveolar bone loss at different teeth sites. Asterisk indicates Kruskal–Wallis test among different age group, *P* < 0.05. **b** Comparison in the proportion of alveolar bone loss between two sexes. **c** Comparison in the proportion of alveolar bone loss among different age groups.

The degree of bone loss differed in the tooth types and sites (*P* < 0.05). Bone loss was more severe in the lateral incisors and less severe in the canines than in the other teeth (Appendix Table 2). This result revealed that the bone loss is significantly larger in mesial-distal sites than in buccal-palatal sites in the incisors, whereas no differences were found among the different sites in the canines (Fig. 1a). The thickness results are as follows: the mean buccal residual thickness: central incisors > lateral incisors > canines and the mean palatal residual thickness: canines > central incisors > lateral incisors. The buccal bone in the incisors was thicker at the apical level, and the palatal thickness tended to increase along the apical direction for all tooth types (Table 2). Among the maxillary anterior teeth, the lateral incisor had the highest incidence of buccal undercut (85.2%) and was closest to the alveolar ridge, while a few teeth presented with a buccal undercut and fenestration in the central incisor. There was a significant difference in the angulation and the apical bone height among the tooth types (*P* < 0.05); the angle in the canines (18.67° ± 6.27°) (95%CI: 17.75–19.59) was larger, and the apical bone height ((9.35 ± 3.01) mm) (95%CI: 8.91–9.79) (95%CI:)was smaller (Table 2).

**Table 2:** Differences in tooth type, sex and age of alveolar bone morphology in the patients with periodontitis.

Alveolar bone morphology	Characteristics											
	Tooth type				Sex			Age				
	Central incisor (*n* = 179)	Lateral incisor (*n* = 176)	Canine (*n* = 180)	*P-*value	Male (*n* = 267)	Female (*n* = 268)	*P-*value	<30 years (*n* = 118)	30–39 years (*n* = 120)	40–49 years (*n* = 149)	≥50 years (*n* = 148)	*P-*value
Alveolar bone loss/ %	29.32 ± 16.19	32.38 ± 18.10	27.02 ± 16.81	<0.001^a*^	29.18 ± 17.06	29.92 ± 17.30	0.352	19.39 ± 13.80	27.07 ± 15.41	34.12 ± 17.21	35.06 ± 16.93	<0.001^a‖^
Buccal bone thickness/mm	1.39 ± 0.37	1.29 ± 0.45	1.13 ± 0.39	<0.001^a†^	1.29 ± 0.40	1.25 ± 0.48	0.044^d^	1.18 ± 0.37	1.25 ± 0.43	1.33 ± 0.41	1.29 ± 0.45	0.030^a¶^
Palatal bone thickness/mm	4.10 ± 0.91	3.34 ± 0.81	4.71 ± 1.15	<0.001^a‡^	4.36 ± 1.07	3.75 ± 1.08	<0.001^d^	4.33 ± 1.05	3.89 ± 1.13	4.10 ± 1.13	3.92 ± 1.11	0.008^a#^
Buccal undercut	78/179 (43.6%)	150/176 (85.2%)	125/180 (69.4%)	<0.001^b*^	153/267 (57.3%)	200/268 (74.6%)	<0.001^b^	83/118 (70.3%)	80/120 (66.7%)	98/149 (68.1%)	92/148 (61.9%)	0.575
Buccal undercut depth/mm	1.56 ± 0.70	1.77 ± 0.75	1.80 ± 0.82	0.071	1.56 ± 0.67	1.87 ± 0.82	<0.001^d^	1.67 ± 0.68	2.06 ± 0.97	1.67 ± 0.66	1.59 ± 0.69	0.006^a**^
Buccal undercut location/mm	5.52 ± 1.70	4.64 ± 1.36	5.82 ± 1.53	<0.001^c*^	5.24 ± 1.57	5.26 ± 1.61	0.827	4.92 ± 1.38	5.63 ± 2.07	5.23 ± 1.47	5.25 ± 1.34	0.102
Buccal fenestration	16/179 (8.9%)	76/176 (43.2%)	85/180 (47.2%)	<0.001^b†^	84/267 (31.5%)	93/268 (34.7%)	0.426	62/118 (52.5%)	40/120 (33.3%)	42/149 (28.2%)	33/148 (22.3%)	<0.001^b‡^
Angulation between long axis of teeth and alveolar process/°	15.09 ± 6.06	14.07 ± 5.99	18.67 ± 6.27	<0.001^c†^	17.26 ± 6.10	14.66 ± 6.46	<0.001^d^	15.71 ± 6.28	15.06 ± 6.43	16.12 ± 6.10	16.71 ± 6.76	0.316
Apical bone height/mm	13.38 ± 2.75	13.15 ± 2.69	9.35 ± 3.01	<0.001^a§^	11.68 ± 3.32	12.21 ± 3.41	0.058	11.12 ± 3.37	11.81 ± 3.22	12.37 ± 2.98	12.30 ± 3.74	0.010^a††^

^a^Kruskal–Wallis test among tooth types: *P* < 0.05

^a*^alveolar bone loss

^a†^buccal bone thickness

^a‡^palatal bone thickness: central incisor versus lateral incisor, *P* < 0.05; central incisor versus canines, *P* < 0.05;

lateral incisor versus canines, *P* < 0.05

^a§^apical bone height: central incisor versus canines, *P* < 0.05; lateral incisor versus canines, *P* < 0.05. Kruskal–Wallis test among age groups: *P* < 0.05

^a‖^alveolar bone loss: <30 years versus 30–39 years, *P* < 0.05; < 30 years versus 40–49 years, *P* < 0.05; <30 years versus ≥50 years, *P* < 0.05; 30–39 years versus 40–49 years, *P* < 0.05; 30–39 years versus ≥50 years, *P* < 0.05

^a¶^buccal bone thickness: <30 years versus 40–49 years and ≥50 years, respectively, *P* < 0.05

^a#^palatal bone thickness: <30 years versus 30–39 years and ≥50 years, respectively, *P* < 0.05

^a**^buccal undercut depth: 30–39 years versus ≥50 years, *P* < 0.05

^a††^apical bone height: <30 years versus 40–49 years and ≥ 50 years, respectively, *P* < 0.05

^b^Chi-square test among tooth types: *P* < 0.05

^b*^the percentage of undercut: central incisor versus lateral incisor, *P* < 0.05; central incisor versus canines, *P* < 0.05; lateral incisor versus canines, *P* < 0.05

^b†^the percentage of fenestration: central incisor versus lateral incisor and canine, respectively, *P* < 0.05. Chi-square test among age groups: *P* < 0.05

^b‡^the percentage of fenestration: <30 years versus 30–39 years, 40–49 years and ≥50 years group, respectively, *P* < 0.05

^c^ANOVA test among tooth types: *P* < 0.05

^c*^buccal undercut location: lateral incisor versus central incisor and canine, respectively, *P* < 0.05

^c†^angulation between long axis of teeth and alveolar process: canine versus central incisor and lateral incisor, respectively, *P* < 0.05

^d^Mann–Whitney *U* test, comparing the morphology between two sexes: *P* < 0.05

#### Sex differences

There were no statistically significant differences in the degree and distribution of bone loss between the two sexes (Fig. 1b). However, males demonstrated a significantly thicker residual bone compared with females (*P* < 0.05). The incidence of buccal undercuts in females who showed a deeper undercut was slightly higher (34.7%) than that in males. No statistically significant difference was measured in apical bone height between the two sexes (Table 2).

#### Age differences

The severity of alveolar bone loss increased with age (*P* < 0.05). Compared with the other groups, the < 30-year-old group revealed more mild bone loss (83.7%), while the proportions of moderate and severe bone loss were higher in the ≥40-year-old group (over 50%) (Fig. 1c). The mean buccal residual thickness was thinner in the <30-year-old group than in the other groups, while the palatal bone was thicker. The proportion of fenestration (52.5%) was the highest in the <30-year-old group. The apical bone height of the ≥40-year-old group was larger than that of the <30-year-old group, and the proportions of moderate and severe bone loss increased with age (Table 2).

#### Sagittal root position in relation to the anterior maxillary alveolar process

**Fig. 2:**
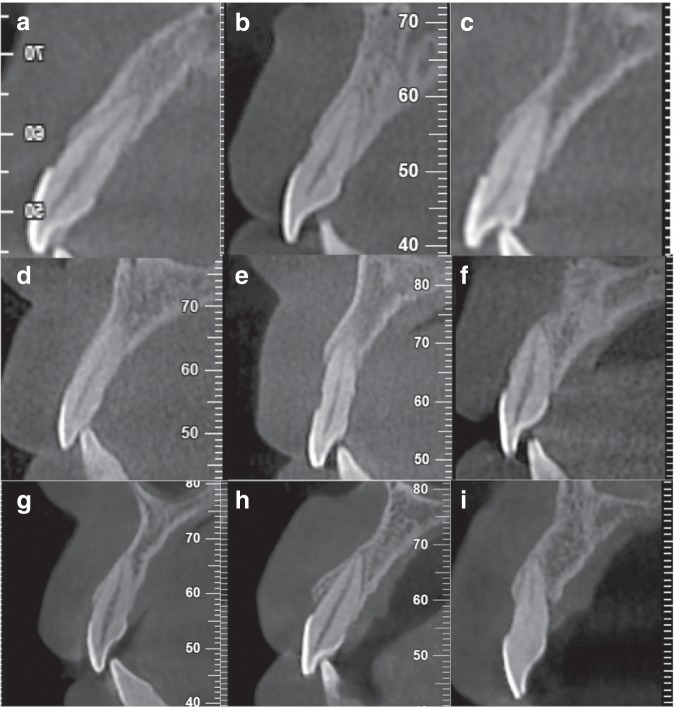
Sagittal root position in relation to the anterior maxillary alveolar process in this study. **a** Type P1**. b** Type B2. **c** Type M2**. d** Type P2. **e** Type N2. **f** Type B3**. g** Type M3. **h** Type P3. **i** Type N3.

Comparing the buccal and palatal bone thicknesses at the mid-root level, the sagittal position of the maxillary anterior teeth in the healthy group and the mild, moderate and severe bone loss groups were mainly type B, accounting for 90.5%, 90.8%, 83.0% and 51.6% of the individuals, respectively; the proportions of type M, P and N gradually increased with the aggravation of bone loss. Comparing the relative position between the long axis of the teeth and the alveolar process, the healthy group and the mild and severe bone loss groups were mainly type 2, the moderate bone loss group was mainly type 3, and only three individuals in the severe bone loss group exhibited type 1 (Table 3). Figure 2 represented the sagittal root position in this study.

**Table 3:** Classification of sagittal root position in the severity of bone loss.

Severity of bone loss	Classification	Number of teeth	Type1	Type2	Type3
No bone loss (*n* = 180)	Type B	163 (90.5%)	0	84 (46.6%)	79 (43.9%)
	Type M	8 (4.5%)	0	3 (1.7%)	5 (2.8%)
	Type P	9 (5%)	0	6 (3.3%)	3 (1.7%)
	Type N	0	0	0	0
Mild bone loss (*n* = 196)	Type B	178 (90.8%)	0	114 (58.2%)	64 (32.6%)
	Type M	7 (3.6%)	0	0	7 (3.6%)
	Type P	11 (5.6%)	0	6 (3.1%)	5 (2.5%)
	Type N	0	0	0	0
Moderate bone loss (*n* = 196)	Type B	162 (82.6%)	0	72 (36.7%)	90 (45.9%)
	Type M	18 (9.2%)	0	11 (5.6%)	7 (3.6%)
	Type P	16 (8.2%)	0	9 (4.6%)	7 (3.6%)
	Type N	0	0	0	0
Severe bone loss (*n* = 143)	Type B	78 (54.5%)	0	44 (30.7%)	34 (23.8%)
	Type M	3 (2.1%)	0	2 (1.4%)	1 (0.7%)
	Type P	36 (25.2%)	3 (2.1%)	22 (15.4%)	11 (7.7%)
	Type N	26 (18.2%)	0	17 (11.9%)	9 (6.3%)

## Discussion

This study measured the mean residual bone thicknesses on the buccal and palatal sides of the maxillary anterior teeth to explore the morphology of alveolar bone in periodontitis patients for clinical guidance use. Gracco et al.^3^ found that alveolar bone thickness was affected by the facial skeletal type and occlusions; thus, our study excluded individuals with malocclusion. Besides, the morphology and quality of alveolar bone would be influenced by diseases meeting with the exclusive criteria, which could generate errors in the process of interpreting CBCT (eg. obscure cemento-enamal junction). Results in the present study show that the mean residual bone in periodontitis patients was thicker on the buccal side, while on the palatal side, it was thinner, which was related to the degree of bone loss. In 1965, Glickman et al.^16^ found that the buttressing bone is formed during the repair of bone loss. Similarly, Hienz et al.^17^ reported that bone loss is often accompanied by compensatory reconstruction. Therefore, there may be compensatory bone formation on the buccal side in periodontitis patients with increased bone destruction and traumatic occlusal forces; however, the morphology of the buttressing bone was irregular.^18^

This study analysed the dimensions of bone thickness in different tooth types, sexes and age subgroups of periodontitis patients, whose distribution was similar to those in a previous study in healthy individuals,^6,19,20^ but there were a few differences between the age subgroups. Our study found that the buccal bone thickness was thinner in the canines than in the other teeth, and the palatal bone was thinner in the lateral incisors than in the other teeth. The major reason for this finding may be the anatomical structure of the root; the canine root is often upright and subjected to larger occlusal forces than the incisors. Our study showed that males demonstrated thicker bone compared with females, which may be mainly related to the difference in skeletal growth. Although the bone thickness changed with the occurrence of periodontitis, to some extent, the distribution of bone thickness did not transform, which may be because the bone in the alveolar crest was first affected by periodontitis.^21^ However, a thinner buccal bone and a thicker palatal bone were found in the <30-year-old group, which was not in agreement with the findings of a previous study. Braut et al.^21^ showed a trend towards decreasing buccal thickness at the crest level with increasing age. This difference may be linked to the history of periodontitis, which needs further exploration of large samples and the degree of bone absorption. The proportion of moderate and severe bone loss increased with age and it was different in terms of race. Periodontal biotype had a moderate association with the underlying bone,^22,23^ and was related to the outcomes of periodontal therapy, implant therapy and root coverage procedures, especially in the aesthetic area of the anterior teeth. Previous studies have shown that the buccal bone should be at least 2 mm to maintain the alveolar bone level;^6,7,24^ however, the mean buccal bone thickness in our study was (1.27 ± 0.42) mm, which was insufficient. To improve the periodontal biotype, reduce bone loss caused by the thinning of the bone and restore aesthetics, we often recommend carrying out bone or soft tissue increment operations^25,26^ before orthodontic treatments or immediate implantations.

Our study analysed the distribution of bone loss by the different tooth types and sites. There was significantly more bone loss in the mesial and distal sides of the incisors than in the other sites. This finding is in agreement with that of Zhao et al.,^11^ which may be related to local anatomical factors and occlusal forces. Halazonetis et al.^27^ believed that subjects with periodontitis exhibited more attachment loss in proximal sites than in other sites, which was due to the structure of the gingiva-col area with non-keratinizing epithelium. Gingiva-col areas are more permeable to bacterial toxins, and it is not easy to control plaque in these areas.^28^ In this study, we found that there were no statistically significant differences in the degree and distribution of bone loss between the two sexes, which was not similar to the findings in previous studies.^9,29^ The difference may be related to the sample population and the distribution by age.

In the present study, the buccal undercut in periodontitis patients was close to the alveolar ridge, and the proportion of undercuts was lower in the severe bone loss group than in the other groups. This finding may be related to the disappearance of undercuts due to buccal bone loss. At present, there are few studies on the quantitative analysis of anterior buccal undercuts. Zhang et al.^19^ analysed the distribution of undercuts among different tooth types in healthy subjects, and the results were similar to ours. Lee et al.^30^ measured the buccal undercut angle in Korean people, finding that the undercuts were obvious when the angle was smaller than 130°. His results showed that a buccal undercut below the root apex of the maxillary central incisor was higher and more curved than other types of tooth. The above difference may be due to differences in the methods of measuring the undercut and the conditions of periodontitis. Zekry et al.^25^ believed that the highest proportion of fenestration is determined at 5 mm below the alveolar crest. Therefore, the reason that the percentage of fenestration was lower in periodontitis patients than in healthy patients may be the presence of excessive bone resorption. In addition, the palatal side of alveolar bone reduced might correlate with vertical bone absorption. Although the buccal bone increased, subsequent implanting or orthodontic plan should be paid more considerations because of the reduction of residual bone height. In this study, the percentage of fenestration was lower in the central incisor than in the other teeth. Evangelista et al.^31^ found that males had more fenestration than females and more fenestration in the incisors. The difference may be related to sample population, occlusion development, and the history of periodontitis. Obvious bone undercuts were often found on the maxillary buccal side and mandible lingual side, increasing the risk of cortical plate perforation and surgical complications^19,32^ in immediate implantation surgery. Although fenestration is common in the maxillary region and is considered a non-pathological condition within the range of periodontal normalcy,^33^ fenestration in periodontitis patients reduces their ability to resist inflammatory infiltration and causes them to be more prone to bone loss. Fenestration sometimes makes the periodontal surgery complicated or changes the implanting plans during the implantation, which needs precise diagnosis.

In the present study, patients with periodontitis had a higher apical bone height than did healthy individuals, and the highest apical bone height occurred in the severe group. This result may be related to pathological buccal-crown displacement with apical bone reactive hyperplasia at the maxillary anterior teeth under occlusal forces. The apical bone height in periodontitis patients varied with the tooth types and age subgroups. This result may be due to the anatomical structure of roots, as the proportions of moderate and severe bone loss increased with age. Monish et al.^34^ found that the apical bone height needs to be at least 3–5 mm and the residual bone height needs to be at least 10 mm to obtain primary stability in immediate implantations. Therefore, it is very important to analyse the specific value of apical bone height in periodontitis patients.

The sagittal root position in relation to the anterior maxillary alveolar process was of great importance for the subsequent immediate implantation in periodontitis patients. Kan et al.^35^ classified the location relationship between root and the bony wall, while they didn’t definite the location between the axis of root and alveolar bone. Laterly Lau et al.^36^ classified the buccal and palatal bone thicknesses at the mid-root level into type B, M, P and classified the angulations of the alveolar processes with respect to the long axis of the root into type 1, 2, and 3. For patients with severe bone loss, the buccal and palatal bone thicknesses at the mid-root level may be “0”; therefore, we added type N to Lau’s classification. The results of our study suggest that most of the positions of the maxillary anterior teeth corresponded to type B, but the proportions of type M, P and N gradually increased with the aggravation of bone loss. These results may be due to irregular bone absorption and reactive buttressing bone formation. The bony characteristics of B1, B2, M2, P2, M3, P3 were similar, to some extent, that the buccal bone was thinner and root oriented toward buccal. Thus, palatal side was recommended in the immediate implantation and guided regenerated surgery could be assisted in occasions. B3 was the most challenging pattern and bone grafting after tooth extraction was recommended to add the success rate.^36^ However, it should be noted that the classification of periodontitis patients in this study was based on the morphology of bone loss; thus, the residual bone height was also a key aspect in the success of implantation. When there was severe bone loss on the palatal side, type B and M may have converted into type P; moreover, the width of residual alveolar bone was reduced. Therefore, we should consider the buccal bone thickness. The shape of the extraction socket in type N had little influence on the immediate implantation due to excessive bone absorption on the buccal and palatal side, so the apical bone height was of great importance for primary stability. And the gingival margin was difficult to recover, and it might be repaired by artificial gingiva.

Maxilla anterior teeth have close relation to patients’ beauty and pronunciation. Recently, an immediate implant with flapless surgery^37-39^ has been shown to reduce the amount of damage to the alveolar ridge and minimize bone resorption by only removing a small amount of tissue from the alveolar crest. However, the morphology of the alveolar bone after resorption cannot be accurately assessed under flapless surgery, which may lead to complications after implantation. Therefore, to obtain a stable implantation, it was very important to accurately evaluate the morphology in periodontitis patients by CBCT before surgery.

As our study was a preliminary study, the sample size was limited; thus, the distribution of alveolar defects should be further explored with a large sample population. At the same time, this study did not classify or summarize some morphological indicators with clinical significance, such as root morphology in the alveolar sockets and the effect of angle on periodontitis prognosis; thus, further analysis is needed to provide a more comprehensive reference for subsequent periodontal treatments.

## Materials and methods

### Subjects

A total of 306 periodontitis patients (mean age 43.46-years-old) and 151 periodontally healthy individuals (mean age 37.07-years-old) were randomly selected from the imaging database at the Affiliated Stomatology Hospital of China Medical University between January 2013 and December 2016. The periodontal disease status was determined according to clinical and CBCT examinations. A total of 90 subjects suffered from classical chronic periodontitis, except for aggressive periodontitis, with bone loss (535 maxilla anterior teeth, mean age: 41.89 years), and 30 healthy individuals (180 maxilla anterior teeth, mean age: 37.33 years) were included with a similar constituent ratio of sexes. The periodontitis and control groups were matched at baseline regarding their demographic (age/sex/tooth type) parameters.The subjects were classified into four categories based on the severity of alveolar bone loss:^11,40^ (a) no bone loss: no radiographic bone loss, the distance from alveolar bone crest to CEJ was 1–2 mm; (b) mild bone loss: radiographic bone loss of <1/3 of the root length (optional); (c) moderate bone loss: radiographic bone loss of 1/3-1/2 of the root length (optional); (d) severe bone loss: radiographic bone loss of å 1/2 of the root length in random sites. The exclusion criteria were as follows: (1) systemic or endocrine diseases that influence bone metabolism (e.g., diabetes, osteoporosis, etc.) (2) local conditions that affect the quality of the bone (e.g. cysts, tumours, prior orthodontics, trauma or surgical history); (3) obvious malocclusion in the maxillary anterior teeth (e.g. moderate and severely deep overbite or overjet, dental crowding, etc.); (4) teeth with prior periodontal treatment, root canal therapy, restoration therapy; (5) periodontitis patients with early and rapid alveolar bone loss.

### CBCT image acquisition

CBCT scans were obtained by NEWTOM VG CBCT (QR-NIM s.r.l.; Verona, Italy) with a field of view (FOV) of 200 mm × 250 mm, a tube voltage of 110 kV and a filament current of 5 mA. The images were acquired by means of NNT software (version 2.19 New Tom), and the data were reconstructed with 0.25 mm thick slices.

### Measurements

**Fig. 3:**
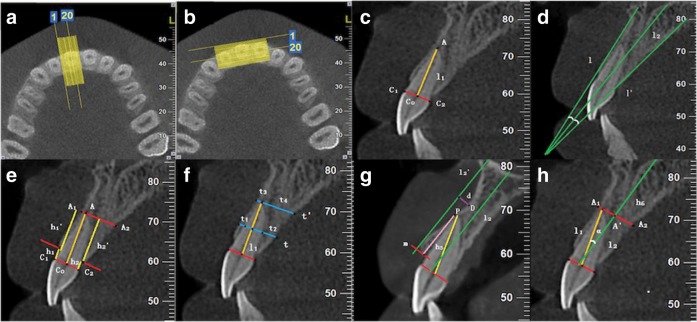
Diagrams of the alveolar morphology measurements. **a** Axial views at the maxillary arch level were perpendicular to the alveolar ridge. **b** Coronal views were parallel to the alveolar ridge. **c** Points C_1_, C_2_, C_0_, and A represent the buccal CEJ point, palatal CEJ point, midpoint of line C_1_C_2_ and apical point. Line C_0_A was the long axis of teeth. **d** Buccal line (line l) and palatal line (line l′) were marked by a line of best fit to the buccal and palatal alveolar surfaces, respectively. The long axis of the alveolar process (line l_2_) was marked by bisecting the line l and l′. **e** Lines perpendicular to the long axis of the teeth through points R and A were drawn. Bone loss was the vertical distance from C to R (buccal, palatal, mesial, and distal sites were h_1_, h_2_, h_3_, and h_4_, respectively), while the residual bone height was measured from R to A (buccal, palatal, mesial, and distal sites were h_1_′, h_2_′, h_3_′ and h_4_′, respectively). **f** Buccal and palatal bone thicknesses were obtained by measuring the distance perpendicular to the long axis of the teeth at the mid-root level (t_1_, t_2_), apical level (t_3_, t_4_) and 1 mm apical to the alveolar crest (t_5_, t_6_). **g** A line parallel to the long axis of the alveolar process and tangent to the buccal cortical bone was drawn. The distance from the deepest point D of the undercut to the line l_2_′ was defined as the buccal undercut depth. A line m perpendicular to the long axis of the alveolar process at point R was drawn. The distance from the buccal undercut convex-concave junction point P to line m was defined as the buccal undercut location. **h** The angulation between l_1_ and l_2_ was the required angle. The apical bone height h_6_ was measured along the long axis of the alveolar process from the root apex to the nasopalatine duct.

The sagittal slices were perpendicular to the alveolar ridge, and the coronal slices were parallel to it. Landmarks were identified and marked in the CT images before the measurements were taken.^27,36^ As shown in Fig. 3, the sagittal slices in which apical points existed were selected. The alveolar ridge point (R), cemento-enamel junction (CEJ) point (C) and apical point (A) were used as reference points. The long axis of the teeth l_1_ and the long axis of the alveolar process l_2_ were identified as reference lines (Fig. 3a–d). Measurements included the level of bone loss, residual bone thickness, buccal undercut location and depth, buccal fenestration, angulation between long axis of teeth and alveolar process, and apical bone height (Fig. 3e–h).^19,41^ The degree of bone loss was described using the percentage of bone loss and was calculated by [(h − 2 mm)/(h + h′ − 2 mm)] × 100%. The overall buccal and palatal residual bone thickness for each tooth was the average thickness of mid-root level, apical level and 1 mm apical to the alveolar crest. Buccal fenestration was considered to exist when root exposure occurred with no buccal bone defects involving the alveolar crest.

### Classification of sagittal root position in relation to the anterior maxillary alveolar process

**Fig. 4:**
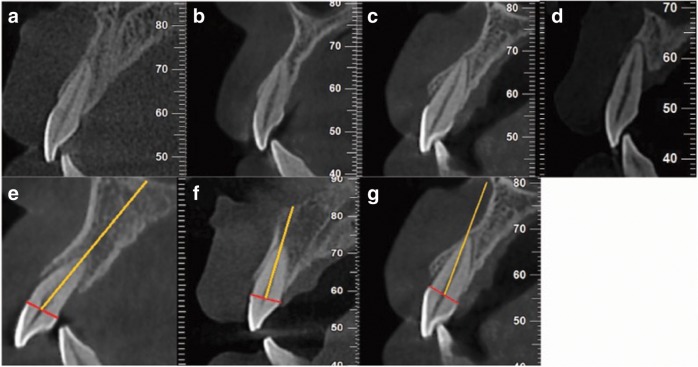
Classification of sagittal root position according to position and angulation. **a** Type B. **b** Type M. **c** Type P. **d** Type N. **e** Type 1. **f** Type 2. **g** Type 3.

Comparing the buccal and palatal bone thickness at the mid-root level, type B: t_1_ < t_2_, t_2_ − t_1_ > 0.1 mm; type M: *t*_1_ ≈ *t*_2_, |*t*_1_ − *t*_2_| < 0.1 mm; type P: *t*_1_ > *t*_2_, *t*_1_ − *t*_2_ > 0.1 mm; and type N: the buccal and palatal residual thickness at the mid-root level was 0 owing to bone loss (Fig. 4a–c).^36^ Comparing the directions of the alveolar processes with respect to the root, type 1: the long axis of the teeth was parallel or in lingual inclination toward the alveolar process; type 2: the long axis of the teeth was slightly inclined toward the buccal bone and located posterior to the most concave point; type 3: the long axis of the teeth was obviously inclined towards the buccal bone and located anterior to the most concave point (Fig. 4d–f).^36^

### Statistical analysis

**Fig. 5:**
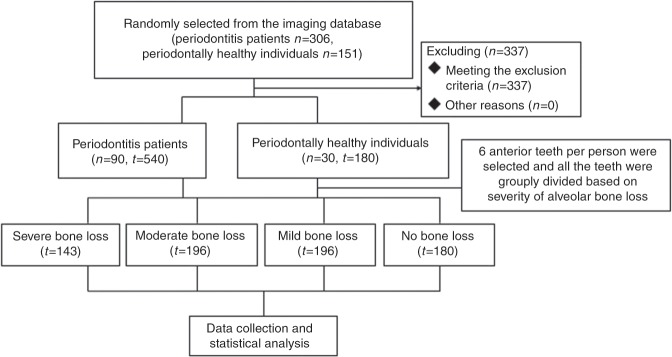
Patient flowchart of the study. N represents number of the individuals and T represents number of the teeth.

A statistical data analysis was performed with SPSS software (Version 24.0; SPSS, Chicago, IL, USA). The Kolmogorov–Smirnov test was used to evaluate the normality of the data. *T*-tests and one-way ANOVA were applied to detect statistically significant differences in normally distributed data, while the Mann–Whitney U and Kruskal–Wallis tests were used for non-normally distributed data. The Friedman test was used to analyse the differences among related samples, and the chi-square test was performed for the frequency analysis. *P* < 0.05 was considered to be statistically significant. A reliability test was used to analyse the variability between the investigators. The kappa value was >0.8 and it had good consistency and accuracy (Fig. 5).

## Conclusions

The morphology of the alveolar bone at the maxillary anterior teeth transformed in periodontitis patients. The buccal residual bone thickness increased significantly when the alveolar bone loss was å 1/2 of the root length in random sites, while the buccal undercut and fenestration decreased. It was difficult to obtain an ideal aesthetic effect for teeth with severe bone loss. The distribution of bone defects may provide guidance for subsequent implants and restorative treatments. Bone or soft tissue increment operations were often recommended before immediate implantation and orthodontic treatment to maintain the bone level and restore aesthetics.

## Supplementary information

Additional document
