# Supplementary material for: The dimension and morphology of alveolar bone at maxillary anterior teeth in periodontitis: a retrospective analysis—using CBCT
Source: Int J Oral Sci. 2020 Jan 14;12:4. doi: 10.1038/s41368-019-0071-0 (PMC6957679; doi:10.1038/s41368-019-0071-0)
Supplement: Supplementary file 1 — Additional document [file 41368_2019_71_MOESM1_ESM.doc]

**SUPPLEMENTARY TABLE 1** Alveolar bone morphology measurements between healthy individuals and periodontitis patients

| Alveolar bone morphology | Healthy individuals （*n*=180） | Periodontitis patients  （*n*=535） | *P*-value |
| --- | --- | --- | --- |
| Buccal bone thickness (mm) | 1.05±0.35 | 1.27±0.42 | 0.000^a^ |
| Palatal bone thickness (mm) | 4.46±1.54 | 4.05±1.12 | 0.005^a^ |
| Buccal undercut (%) | 112/180(67.8%) | 353/535(66.0%) | 0.360 |
| Buccal undercut depth (mm) | 1.56±0.65 | 1.74±0.77 | 0.053 |
| Buccal undercut location (mm) | 5.85±1.46 | 5.25±1.59 | 0.000^a^ |
| Buccal fenestration (%) | 87/180(48.3%) | 177/535(34.2%) | 0.000^b^ |
| Angulation between long axis of teeth and alveolar process (°) | 17.21±6.90 | 15.96±6.41 | 0.035^a^ |
| Apical bone height (mm) | 10.17±3.17 | 11.95±3.37 | 0.000^a^ |

***n***: Number of teeth

**a**: Mann-Whitney U test, comparing healthy individuals and periodontitis patients: *P*<0.05

**b**: Chi-square test, comparing healthy individuals and periodontitis patients: *P*<0.05

**SUPPLEMENTARY TABLE 2** Differences in buccal and palatal residual bone thickness among different positions

|  | Buccal residual bone thickness | | |  | | Palatal residual bone thickness | | |  |
| --- | --- | --- | --- | --- | --- | --- | --- | --- | --- |
| Tooth | At 1mm from alveolar crest | Mid-root level | Apical level | *P-*value | At 1mm from alveolar crest | | Mid-root level | Apical level | *P-*value |
| Central incisor（*n*=179） | 1.03±0.33 | 1.12±0.43 | 1.97±0.75^*^ | 0.000 | 1.59±0.51^*^ | | 2.63±0.91^*^ | 7.88±1.95^*^ | 0.000 |
| Lateral incisor（*n*=176） | 1.10±0.36 | 1.07±0.54 | 1.63±0.99^*^ | 0.000 | 1.31±0.35^*^ | | 1.95±0.81^*^ | 6.48±1.77^*^ | 0.000 |
| Canine（*n*=180） | 1.14±0.42 | 1.12±0.62 | 1.14±0.95 | 0.461 | 1.51±0.45^*^ | | 2.71±0.86^*^ | 9.83±2.77^*^ | 0.000 |

***n***: Number of teeth

***:** Friedman test, comparing among different positions: *P*<0.05
